# The Impact of COVID-19 on Sleep Quality in People Living With Disabilities

**DOI:** 10.3389/fpsyg.2021.786904

**Published:** 2021-12-23

**Authors:** Nikki Heinze, Syeda F. Hussain, Claire L. Castle, Lauren R. Godier-McBard, Theofilos Kempapidis, Suzanne Ftouni, Colin A. Espie, Renata S. M. Gomes

**Affiliations:** ^1^BRAVO VICTOR, Research, London, United Kingdom; ^2^Research and Innovation, Blind Veterans UK, London, United Kingdom; ^3^Veterans and Families Institute for Military Social Research, Anglia Ruskin University, Chelmsford, United Kingdom; ^4^Circadian Therapeutics, Oxford, United Kingdom; ^5^Nuffield Department of Clinical Neurosciences, Sleep & Circadian Neuroscience Institute (SCNi), University of Oxford, Oxford, United Kingdom; ^6^Northern Hub for Veterans and Military Families Research, Department of Nursing, Midwifery and Health, Faculty of Health and Life Sciences, Northumbria University, Newcastle, United Kingdom

**Keywords:** sleep quality, PSQI, disability, visual impairment, sight loss, COVID-19

## Abstract

**Background:** Research exploring the impact of the COVID-19 pandemic on sleep in people with disabilities has been scarce. This study provides a preliminary assessment of sleep in people with disabilities, across two timepoints during the pandemic, with a focus on those with visual impairment (VI).

**Methods:** Two online surveys were conducted between April 2020 and March 2021 to explore sleep quality using the Pittsburgh Sleep Quality Index (PSQI). A convenience sample of 602 participants completed the first survey and 160 completed the follow-up survey.

**Results:** Across both timepoints, participants with disabilities reported significantly poorer global sleep quality and higher levels of sleep disturbance, use of sleep medication and daytime dysfunction than those with no disabilities. Participants with VI reported significantly higher levels of sleep disturbance and use of sleep medication at both timepoints, poorer global sleep quality, sleep duration and latency at time 1, and daytime dysfunction at time 2, than those with no disabilities. Global sleep quality, sleep duration, sleep efficiency, and self-rated sleep quality deteriorated significantly in participants with no disabilities, but daytime dysfunction increased in all three groups. Disability and state anxiety were significant predictors of sleep quality across both surveys.

**Conclusion:** While sleep was consistently poorer in people with disabilities such as VI, it appears that the COVID-19 pandemic has had a greater impact on sleep in people with no disabilities. State anxiety and, to a lesser extent, disability, were significant predictors of sleep across both surveys, suggesting the need to address anxiety in interventions targeted toward improving sleep.

## Introduction

The COVID-19 pandemic has impacted people’s lifestyles and routines worldwide. In the initial absence of a vaccine, governments across the globe introduced measures such as mask-wearing, social distancing, shielding, self-isolation, and quarantining to reduce the spread of the coronavirus. A number of countries, including the United Kingdom, introduced government-mandated “lockdowns,” which limited movement and social contact. Unsurprisingly, there has been a growing focus on the mental and physical health impacts of these restrictions, including those relating to sleep (Groarke et al., 2020; Pérez-Carbonell et al., 2020; Killgore et al., 2021). Poor sleep has been associated with poorer health-related quality of life (Lo and Lee, 2012), diminished cognitive functioning, and poor mental health, including increased incidence of anxiety and depression (Benitez and Gunstad, 2012; Gadie et al., 2017; Ingram et al., 2020). Changes in sleep duration have also been linked to increased alcohol consumption (Neill et al., 2020) and long-term health effects, including the incidence of physical health conditions such as hypertension, activation of the sympathetic nervous system, impaired glucose control, and increased inflammation (Alvarez and Ayas, 2004).

Research suggests that self-reported sleep patterns, sleep duration, and sleep quality have all worsened under lockdown (Gupta et al., 2020; Pérez-Carbonell et al., 2020). A study conducted in the United Kingdom in May 2020 found that 50% of participants reported that their sleep had been disrupted more than usual, 39% reported that they had been sleeping fewer hours per night compared to before the lockdown, and 29% reported sleeping longer hours but feeling less rested (King's College London and IPSOS Mori, 2020). One proposed reason for the observed impact of the pandemic on sleep is disruption to circadian rhythms, as a result of increased time spent indoors and, consequently, less daylight exposure (Cardinali et al., 2020; Morin et al., 2020). Lifestyle and lifestyle changes prompted by the pandemic, such as disruption to daily physical activity, but not low levels of physical activity (Diniz et al., 2020; Gupta et al., 2020), and alcohol consumption (Romero-Blanco et al., 2020; Robillard et al., 2021), but not changes in alcohol consumption (Ingram et al., 2020), have been found to impact sleep during the pandemic. Anxiety, depression, and stress brought on by the pandemic may have further contributed to irregular sleep patterns (Altena et al., 2020; Evans et al., 2021; Robillard et al., 2021; Villadsen et al., 2021). Research from China found that stress and anxiety were associated with poorer sleep quality in people with lower levels of social capital (i.e., a sense of trust, belonging, and participation within society) who were self-isolating for 14 days at the beginning of the pandemic (Xiao et al., 2020). In addition, chronic illnesses such as hypertension, diabetes, and arthritis have also been linked to sleep difficulties during the pandemic (Robillard et al., 2021). In the United Kingdom, those most at risk due to underlying health conditions were instructed to “shield,” which meant no social contact for long periods of time. Shielding has been associated with poor sleep, with more people in the shielding group than expected experiencing poorer sleep (Ingram et al., 2020). One factor at play may be loneliness, which has previously been found to have a reciprocal effect on sleep; higher levels of loneliness correlate with higher levels of disturbed sleep (Griffin et al., 2020; Groarke et al., 2020). Indeed, loneliness has been identified as a contributing factor in clinical insomnia during the pandemic (Kokou-Kpolou et al., 2020).

Visual impairment (VI) typically refers to reduced vision, which is not correctable with glasses, contact lenses, or surgery, and can range from mild to severe vision loss or blindness. Blind people with no light perception may be at particular risk of poor sleep quality and short sleep duration (Leger et al., 1999; Peltzer and Phaswana-Mafuya, 2017; Hartley et al., 2018). This may be a result of disruption to circadian rhythms, responsible for the sleep-wake cycles, due to a reduction or complete lack of light information being relayed from the eye to the master circadian clock in the hypothalamus (Lockley et al., 2007). Research conducted prior to the pandemic found a higher incidence of disrupted sleep, greater sleep latency, shorter sleep duration, greater daytime disruption, irregular sleep patterns, and difficulty maintaining sleep in people living with VI, particularly those with no light perception, compared to controls (Tabandeh et al., 1998; Tamura et al., 2016). Poor sleep has also been found in people living with other types of disability such as intellectual and developmental disabilities (Richdale and Baker, 2014), hearing difficulties (Test et al., 2011), and traumatic brain injuries (Castriotta et al., 2007; Ouellet et al., 2015).

Very little research has considered the impact of the pandemic on sleep in people with disabilities such as VI, although the existing evidence indicates that disability may have impacted on sleep at this time. One study reported that people living with a disability were more likely to report shorter sleep duration (<6 h) than those without a disability (Pérez-Carbonell et al., 2020), and another reported a high prevalence of insomnia (71%) in those with disabilities, including VI (Necho et al., 2020). Given the impact of sleep on mental and physical health, it is important to understand how the pandemic has affected the sleep quality of those living with disabilities such as VI. The present paper sets out to explore sleep quality in people with disabilities, with a focus on those living with VI, as the pandemic progressed.

## Materials and Methods

Longitudinal data were collected in two online surveys conducted first between March and April 2020 (T1) and a follow-up conducted in March 2021 (T2). Additional details of methods and findings relating to the sample used in this study are reported in Heinze et al. (2021) and are available to supplement the findings in this article.

### Materials

The online survey was developed in Microsoft Forms (Microsoft Corporation, Redmond, WA) by the Research and Innovation team at Blind Veterans UK, a charity supporting British veterans with sight loss, in collaboration with the University of Oxford. The survey platform was selected due to its accessibility features for participants with VI including compatibility with screen readers, color contrast, and high contrast settings. The survey was further made accessible by splitting grid questions across individual pages, so that participants were shown only one question per page to ensure ease of reading.

In addition to participant information, consent, and demographics, the questionnaire consisted of four sections: current life circumstances (e.g., employment and self-isolation status); health and health behaviors (e.g., disability, alcohol consumption); sleep quality; and social well-being (loneliness, anxiety). The questionnaire was amended for T2 to improve data quality (examples given below) and reduce participant burden.

#### Disability Status

At T1, disability status was assessed by first asking participants if they had a disability followed by a question which instructed them to select all types of disability that applied to them from a list of 16 conditions including “VI or blindness” (see Table 1). At T2, participants were asked if they considered themselves to have a disability followed by a grid question which required them to select “Yes,” “No,” or “Prefer not to say” for each of the 16 conditions. As a result, the mean number of conditions reported increased from 2 at T1 to 3 at T2. As many as six additional participants reported having conditions such as disability affecting mobility or mental health conditions at T2. However, one person indicated having limb loss at T1 but not T2.

**Table 1:** Prevalence of disability and types of disabilities at T1 and T2.

*N*		T1% (*n*)	T2% (*n*)
			602	160
Has a disability	Yes	33.7 (203)	33.1 (53)
Type of disability
	Hearing impairment or deafness	10.0 (60)	11.3 (18)
	Acquired brain injury	4.0 (24)	4.4 (7)
	Limb loss	0.5 (3)	0.6 (1)
	I am immunocompromised	1.5 (9)	3.1 (5)
	Multiple sclerosis	1.2 (7)	1.3 (2)
	Disability affecting mobility	9.5 (57)	16.3 (26)
	Medical conditions (i.e., epilepsy, asthma, and diabetes)	8.8 (53)	12.5 (20)
	Emotional/behavioral difficulties	2.3 (14)	5.6 (9)
	Mental Health issues	10.3 (62)	13.1 (21)
	Temporary disability after illness/accident	0.7 (4)	0.6 (1)
	Profound complex disabilities	1.3 (8)	1.9 (3)
	Learning difficulties	0.3 (2)	2.5 (4)
	Dyslexia	1.5 (9)	0.6 (1)
	Dyscalculia	0.3 (2)	-
	Dyspraxia	1.5 (9)	-	VI or blindness	22.9 (138)	23.1 (37)
VI	VI only	34.1 (47)	24.3 (9)
	VI and comorbid conditions	65.9 (91)	75.7 (28)

% = proportion of the sample who reported having a disability/the condition, *n* = number of participants who reported having a disability/the condition.

#### Self-Isolation

Self-isolation status was assessed with a single question which asked participants to indicate how long they had been self-isolating from a list of response options which included “I am a keyworker/not able to self-isolate” and “I do not self-isolate” and ranged from “0–2 weeks” to “Over 12 weeks” at T1 and from “0–2 weeks” to “Over 6 months” at T2.

#### Alcohol Consumption

At T1, alcohol consumption was assessed by two questions asking participants if they drank alcohol, followed by how often they had been drinking alcohol, with response options ranging from “Once a week” to “Every day.” At T2, the questions were combined into a single question which asked how often participants had been drinking alcohol over the last 3 weeks and which included the response option “I do not drink alcohol.”

#### State Anxiety

State anxiety was assessed using the 20-item state anxiety subscale (STAI-S) of the State Trait Anxiety Index (Spielberger, 1970, 1983). Only state anxiety, as opposed to trait anxiety, was measured in this study. In addition to ensuring brevity of the survey, this was to determine current feelings of anxiety at different timepoints during the pandemic, instead of an individual’s proclivity to experience anxiety. The STAI-S consists of 10 positively and 10 negatively worded statements. Respondents are instructed to indicate how they are feeling “right now” on a scale of 1 (*Not at all*) to 4 (*Very much*). Positively worded items are reverse-scored, and all scale responses are summed to derive a subscale score ranging from 20 to 80, with higher scores indicative of greater state anxiety. Spielberger et al. (1983) reported excellent internal validity of the STAI-S with a median alpha coefficient of *α* = 0.93 (ranging from *α* = 0.86 to 0.95) for samples of working-age adults, college students, high school students, and military recruits and relatively poor test–retest reliability with a median correlation of *r* = 0.33 (ranging from *r* = 0.16 to *r* = 0.62) for samples of college and high school students ascribed to the temporary nature of state anxiety.

#### Loneliness

Loneliness was assessed using version 3 of the UCLA Loneliness scale (Russell, 1996). The scale consists of 20 items that measure self-reported feelings of loneliness and social isolation. Scale responses are summed to generate a loneliness score ranging from 20 to 80, with higher scores indicative of higher levels of loneliness. Russell (1996) reported high internal validity for different sample populations ranging from *α* = 0.89 (in samples of elderly and teachers) to *α* = 0.94 (in a sample of nurses) and a test–retest reliability over 1 year of *r* = 0.73 for a sample of elderly.

#### Sleep Quality

Sleep quality over the last month was assessed using the Pittsburgh Sleep Quality Index (PSQI; Buysse et al., 1989). The PSQI is a self-report measure consisting of 19 items, which are used to derive seven component scores (self-reported sleep quality, sleep latency, sleep duration, sleep efficiency, sleep disturbance, use of sleep medication, and daytime dysfunction). The component scores are summed to derive a global PSQI score ranging from 0 to 21, with higher scores indicating worse sleep quality. Buysse et al. (1989) reported an internal consistency of *α* = 0.83 for the PSQI and test–retest reliability of *r* = 0.87 for the global PSQI score. Respondents with a global PSQI score of >5 are categorized as poor sleepers (sleep outcome). Sleep outcome has a diagnostic sensitivity of 89.6% and specificity of 86.5% for distinguishing between good and poor sleepers (Buysse et al., 1989).

### Recruitment

Data collection for T1 took place between April 1, 2020, and May 15, 2020. A convenience sample was recruited through the researchers’ personal and professional networks, social media, and professional forums. Participants who had consented to being recontacted and had provided a valid email address were invited to take part in T2. Data collection for T2 took place between March 8, 2021 and March 28, 2021. A small number of participants (*n* < 9) across both timepoints were unable to complete the questionnaire by themselves and instead completed it over the telephone with a researcher reading out the questions and entering the responses given.

### Procedure

The Medical Sciences Interdivisional Research Ethics Committee at the University of Oxford advised that ethical approval was not required for this study. Participants accessed the survey via a clickable link embedded in the study invitation. At the start of both surveys, participants were provided with detailed information about the study objective and their rights as research participants. Participants were then asked to provide informed consent to take part in the research by agreeing or disagreeing to a list of consent statements. Participants were able to select if they wanted to answer or skip each of the four main sections, and “Prefer not to say” options were given at most questions. At the end of T1, participants were asked to provide contact details if they consented to being re-contacted for follow-up research.

### Analysis

Duplicates and records without responses were removed from the dataset before analysis. Responses were treated as missing and excluded from the analysis where participants had chosen to skip a section, selected “Prefer not to say,” had not responded to a survey item, or had responded “Other” to questions on sleep disturbance and time taken to fall asleep (this option was included to account for non-normative experiences such as those associated with being bedridden). No global PSQI score was calculated for participants with a missing component score. Proportions were calculated based on the total number of participants giving a valid response at a question excluding those who selected “Prefer not to say” or skipped the question.

Due to a typographical error, the STAI-S scale item Q4 was presented with an incorrect adjective at T1. This was corrected for T2. As a result of this error, a revised anxiety score was calculated for both surveys, which excluded the incorrect item Q4, Cronbach’s *α* = 0.96 for T1 and T2, respectively. The revised scores were used for descriptive statistics for T1 and T2 and regression analyses.

The aim of this study was to assess sleep quality in individuals with disabilities in general, with a focus on those living with VI. Subgroup analysis therefore initially compared participants who reported having one or more types of disability (including those who reported having VI) to participants who reported having no disabilities, and then participants who reported having “VI or blindness” to those who reported no disabilities. Due to small sample sizes in T2 (nine participants reported VI only), it was not possible to control for other disabilities in the VI group. Thus, the group reporting any type of disability included participants with VI and without VI, and the VI group contained participants with comorbid disabilities.

Global PSQI sleep scores were not normally distributed for the three subgroups, as assessed by Shapiro–Wilk’s test (*p* < 0.05). As a result, nonparametric tests were used to assess between- and within-group differences, and medians and interquartile ranges (IQR) are reported in addition to means and standard deviations (SD).

Analysis sought to address three questions:

1. If and how participants with any type of disability and those with VI differed from participants with no disabilities at the two timepoints. To address this, descriptive statistics including mean and SD as well as median and IQR are reported for participants with one or more disabilities, participants with VI and participants with no disabilities; Chi-square tests were used to assess sleep outcome in participants with one or more disabilities vs. participants with no disabilities and participants with VI vs. participants with no disabilities. Mann–Whitney U tests were used to assess between-group differences in global PSQI scores and PSQI component scores between participants with one or more disabilities vs. participants with no disabilities and participants with VI vs. participants with no disabilities.
2. If and how sleep quality changed between the two surveys within each subgroup. Wilcoxon signed-rank or sign tests were used to explore within-group differences between T1 and T2 global PSQI scores and PSQI component scores in participants with one or more disabilities, participants with VI and participants with no disabilities, respectively.
3. What factors predicted sleep quality at both timepoints, and, in particular, whether disability predicted sleep quality when controlling for other factors. A hierarchical linear regression was conducted at T1 and repeated at T2 to identify consistent factors.

## Results

### Participant Characteristics

Table 2 provides an overview of participant characteristics in both surveys. After removing duplicates and surveys which yielded no responses, a total of 602 participants completed T1. The majority of these were white, male, aged 46–55, and in paid employment. Participants resided in 22 different countries, predominantly the United Kingdom. The majority of participants had been self-isolating for 2–4 weeks and were not drinking alcohol. Mean loneliness was 42.54 (*SD* = 13.91), and mean state anxiety using the revised score was 40.52 (*SD* = 13.87). Full results for loneliness have been reported elsewhere (Heinze et al., 2021), and manuscripts reporting results for health behaviors (including alcohol consumption and self-isolation) and state anxiety have been submitted for publication.

**Table 2:** Sample characteristics at T1 and T2.

		T1 % (*n*)	T2 % (*n*)
Gender	Female	47.7 (285)	52.2 (83)
	Male	52.3 (312)	47.8 (76)
Age	18–25	3.0 (17)	1.9 (3)
	26–35	13.3 (76)	11.4 (18)
	36–45	21.6 (123)	17.7 (27)
	46–55	28.4 (162)	31.0 (49)
	56–65	20.7 (118)	24.1 (38)
	66–75	8.8 (50)	10.8 (17)
	76–85	3.5 (20)	3.2 (5)
	86+	0.7 (4)	-
Ethnicity	Asian	1.9 (11)	1.3 (2)
	Black/African/Caribbean	1.9 (11)	0.6 (1)
	Hispanic, Latino or Spanish origin	0.8 (5)	1.9 (3)
	Mixed/multiple ethnic groups	2.5 (15)	1.3 (2)
	White or other White	92.6 (550)	95.0 (152)
	Other	0.3 (2)	-
Country of residence^1^	United Kingdom	61.9 (372)	76.9 (123)
	United States	16.8 (101)	9.4 (15)
	Portugal	10.3 (62)	3.1 (5)
	Malta	3.5 (21)	5.6 (9)
	Germany	1.3 (8)	1.9 (3)
	France	1.2 (7)	1.3 (2)
	Other^2^	5.0 (30)	1.9 (3)
Employment status	In paid employment	68.0 (372)	69.6 (110)
	I am employed but furloughed	-	1.3 (2)
	Retired	22.7 (124)	17.7 (28)
	Unemployed and not looking for work	6.2 (34)	9.5 (15)
	Unemployed but looking for work	3.1 (17)	1.9 (3)
Time spent self-isolating	I’m not self-isolating	26.7 (158)	70.9 (112)
	0–2 weeks	5.6 (33)	0.6 (1)
	2–4 weeks	37.2 (220)	0.0 (0)
	4–8 weeks	27.2 (161)	0.6 (1)
	8–12 weeks	1.4 (8)	1.3 (2)
	Over 12 weeks (T1)/3–4 months (T2)	2.0 (12)	0.6 (1)
	4–5 months (T2 only)	N/A	1.3 (2)
	Over 6 months (T2 only)	N/A	24.7 (39)
Alcohol consumption	I do not drink alcohol	35.9 (207)	35.7 (56)
	Once a week	13.5 (78)	15.9 (25)
	Only on weekends	14.1 (81)	19.1 (30)
	3–5 times a week	26.4 (152)	25.5 (40)
	Every day	10.1 (58)	3.8 (6)

1 The “Country of residence” question was not repeated at T2. Frequencies and proportions reported at T2 are based on responses given at T1.

2 At T1, the “Other” category includes six participants residing in Greece, 3, respectively, in Canada, Cyprus, South Africa, and Sweden, two in Puerto Rico, and one, respectively, in Argentina, Australia, Ireland, Israel, Kenya, Netherlands, Pakistan, Philippines, Switzerland, and Thailand. At T2, the “Other” category includes one participant, respectively, residing in Canada, Greece, and Thailand.

% = proportions of participants giving this response out of the total of valid responses received for this question (excluding “Prefer not to say” and missing responses), *n* = number of participants who selected this response. The total of valid responses achieved for each question excludes those who selected “Prefer not to say” or did not provide a response at this question and can be calculated by summing the number of participants listed for each response option at this question.

In total, 329 T1 participants were invited to take part in T2, 163 yielded responses (49.5% response rate). After removing cases who did not wish to take part in the research (*n* = 2) and duplicates (*n* = 1), a total of 160 individuals completed T2. There were no statistically significant differences between T1 participants who were invited to but did not complete T2 and those who completed T2 in terms of sex, age group, ethnicity, continent of residence (which was compared due the small numbers resident in countries outside of the United Kingdom), and employment status. The majority of T2 participants were white, female, aged 46–55, and in paid employment. One participant had lost their job during COVID-19 and was currently looking for work. Participants resided in nine different countries, the majority in the UK. The smaller sample likely resulted in reduced global distribution of participants compared to T1. The majority of participants were not self-isolating and did not drink alcohol. Mean loneliness was 42.18 (*SD* = 14.54), and mean state anxiety using the revised STAI score was 38.08 (*SD* = 14.27).

#### Disability and VI

Around two-thirds of participants in both surveys reported no disabilities and around a third reported having one or more types of disability (Table 1) with a maximum of eight distinct types of disability being reported by one participant. The most common disability at both timepoints was VI, followed by mental health issues, hearing impairment, and disability affecting mobility at T1, and disability affecting mobility, mental health issues, and medical conditions such as asthma, diabetes or epilepsy at T2. It should be noted that the prevalence of VI in both surveys is unsurprising considering the survey was sent to members of Blind Veterans UK and contacts within the sight loss sector. Among participants with VI, comorbidity was high at both timepoints, the most commonly reported comorbid conditions being hearing impairment (36%) and medical conditions (24%) at T1 and disability affecting mobility (48.6%) and hearing impairment (43.2%) at T2.

### Group Differences in Sleep Quality

Overall, sleep quality over the past month was poor at both timepoints, particularly among those with disabilities. Participants with disabilities scored significantly poorer on median global sleep quality than those with no disabilities at both timepoints (Table 3). At the time of T1 (April–May 2020), 71.8% of participants with disabilities were categorized as having poor sleep (a global PSQI score of >5) compared to 56.3% of participants with no disabilities, *χ*^2^(1, *N* = 545) = 12.10, *p* < 0.01, Cramer’s V = 0.149. Similar proportions of poor sleepers were found at T2 (March 2021), with 69.4% of participants with disabilities being categorized as having poor sleep compared to 57.3% of participants with no disabilities. However, this was no longer significantly different, *χ*^2^(1, *N* = 152) = 2.05, *p* = 0.152. In addition, participants with disabilities also scored significantly poorer on all seven PSQI components at T1, but by T2, only sleep disturbance, use of sleep medication, and daytime dysfunction remained significantly poorer.

**Table 3:** Between-group comparison of Pittsburgh Sleep Quality Index (PSQI) global and component scores for 1+ disabilities and no disability subgroups.

		T1			T2		
		No disability	1+ disabilities	Mann-Whitney U test	No disability	1+ disabilities	Mann-Whitney U test
PSQI global score	*M* (*SD*)	6.64 (3.84)	9.28 (5.03)	*U* = **42,353**, *p* < **0.001**	7.04 (3.82)	9.73 (5.29)	*U* = **3,244.5**, *p* < **0.01**
	*Mdn* (*IQR*)	6.00 (5)	9.00 (9)		7.00 (6)	8.00 (10)	
Sleep duration	*M* (*SD*)	0.39 (0.75)	0.89 (1.11)	*U* = **45,998.5**, *p* < **0.001**	0.55 (0.83)	0.98 (1.19)	*U* = 3,037.5, *p* = 0.057
	*Mdn* (*IQR*)	0.00 (1)	0.00 (2)		0.00 (1)	0.00 (2)	
Sleep efficiency	*M* (*SD*)	1.36 (1.34)	1.66 (1.35)	*U* = **40,624.5**, *p* < **0.05**	1.51 (1.25)	1.90 (1.34)	*U* = 3,017, *p* = 0.088
	*Mdn* (*IQR*)	1.00 (3)	2.00 (3)		1.00 (3)	3.00 (3)	
Sleep latency	*M* (*SD*)	1.20 (1.02)	1.63 (1.14)	*U* = **40,964**, *p* < **0.001**	1.09 (1.03)	1.45 (1.14)	*U* = 3,157.5, *p* = 0.058
	*Mdn* (*IQR*)	1.00 (2)	2.00 (2)		1.00 (2)	1.00 (2)	
Sleep disturbance	*M* (*SD*)	1.24 (0.57)	1.58 (0.73)	*U* = **44,458.5**, *p* < **0.001**	1.32 (0.51)	1.76 (0.76)	*U* = **3,480**, *p* < **0.001**
	*Mdn* (*IQR*)	1.00 (1)	2.00 (1)		1.00 (1)	2.00 (1)	
Sleep quality	*M* (*SD*)	1.14 (0.75)	1.45 (0.92)	*U* = **43,334**, *p* < **0.001**	1.20 (0.80)	1.39 (0.80)	*U* = 3,018, *p* = 0.160
	*Mdn* (*IQR*)	1.00 (1)	1.00 (1)		1.00 (1)	1.00 (1)	
Use of sleep medication	*M* (*SD*)	0.39 (0.94)	0.98 (1.33)	*U* = **44,442**, *p* < **0.001**	0.34 (0.82)	0.94 (1.33)	*U* = **3,154.5**, *p* < **0.01**
	*Mdn* (*IQR*)	0.00 (0)	0.00 (3)		0.00 (0)	0.00 (3)	
Daytime dysfunction	*M* (*SD*)	0.97 (0.69)	1.31 (0.89)	*U* = **44,468**, *p* < **0.001**	1.01 (0.65)	1.47 (0.92)	*U* = **3,413.5**, *p* < **0.01**
	*Mdn* (*IQR*)	1.00 (0)	1.00 (1)		1.00 (0)	1.00 (1)	

*M*, mean score; *SD*, standard deviation; *Mdn*, median score; and *IQR*, interquartile range. Statistically significant results are shown in bold.

Poor sleep was also more prevalent among participants with VI compared to those with no disabilities, but this was not statistically significant at either timepoint; 63.2% of those with VI were categorized as having poor sleep at T1, *χ*^2^(1, *N* = 485) = 1.78, *p* = 0.182, and 63.6% at T2, *χ*^2^(1, *N* = 136) = 0.42, *p* = 0.519. While mean global sleep quality was also worse in those with VI at both timepoints, median sleep quality was significantly worse at T1 only (Table 4). Compared to those with no disabilities, participants with VI reported significantly more disturbed sleep and use of sleep medication at both timepoints, in addition to shorter sleep duration and greater sleep latency at T1, and increased daytime dysfunction at T2.

**Table 4:** Between-group comparison of PSQI global and component scores for visual impairment (VI) and no disability subgroups.

		T1			T2		
		No disability	VI	Mann-Whitney U test	No disability	VI	Mann-Whitney U test
PSQI global score	*M* (*SD*)	6.64 (3.84)	8.15 (4.94)	*U* = **24,833**, *p* < **0.05**	7.04 (3.82)	8.61 (5.06)	*U* = 1,967, *p* = 0.173
	*Mdn* (*IQR*)	6.00 (5)	7.00 (8)		7.00 (6)	7.00 (8)	
Sleep duration	*M* (*SD*)	0.39 (0.75)	0.77 (1.04)	*U* = **29,169**, *p* < **0.001**	0.55 (0.83)	0.82 (1.09)	*U* = 1,960.5, *p* = 0.277
	*Mdn* (*IQR*)	0.00 (1)	0.00 (1)		0.00 (1)	0.00 (2)	
Sleep efficiency	*M* (*SD*)	1.36 (1.34)	1.36 (1.34)	*U* = 23,948.5, *p* = 0.967	1.51 (1.25)	1.79 (1.43)	*U* = 1,960.5, *p* = 0.314
	*Mdn* (*IQR*)	1.00 (3)	1.00 (3)		1.00 (3)	3.00 (3)	
Sleep latency	*M* (*SD*)	1.20 (1.02)	1.45 (1.13)	*U* = **25,284.5**, *p* < **0.05**	1.09 (1.03)	1.20 (1.08)	*U* = 1,942.5, *p* = 0.595
	*Mdn* (*IQR*)	1.00 (2)	1.00 (3)		1.00 (2)	1.00 (2)	
Sleep disturbance	*M* (*SD*)	1.24 (0.57)	1.50 (0.71)	*U* = **28,198.5**, *p* < **0.001**	1.32 (0.51)	1.66 (0.76)	*U* = **2,226**, *p* < **0.05**
	*Mdn* (*IQR*)	1.00 (1)	1.00 (1)		1.00 (1)	1.00 (1)	
Sleep quality	*M* (*SD*)	1.14 (0.75)	1.27 (0.89)	*U* = 26,020.5, *p* = 0.233	1.20 (0.80)	1.26 (0.78)	*U* = 1,894.5, *p* = 0.762
	*Mdn* (*IQR*)	1.00 (1)	1.00 (1)		1.00 (1)	1.00 (1)	
Use of sleep medication	*M* (*SD*)	0.39 (0.94)	0.98 (1.32)	*U* = **29,560.5**, *p* < **0.001**	0.34 (0.82)	0.82 (1.29)	*U* = **2,071.5**, *p* < **0.05**
	*Mdn* (*IQR*)	0.00 (0)	0.00 (3)		0.00 (0)	0.00 (2)	
Daytime dysfunction	*M* (*SD*)	0.97 (0.69)	1.13 (0.87)	*U* = 26,224.5, *p* = 0.102	1.01 (0.65)	1.34 (0.91)	*U* = **2,204**, *p* < **0.05**
	*Mdn* (*IQR*)	1.00 (0)	1.00 (1)		1.00 (0)	1.00 (1)	

*M*, mean score; *SD*, standard deviation; *Mdn*, median score; and *IQR*, interquartile range. Statistically significant results are shown in bold.

### Changes in Sleep Quality Over Time

Global PSQI scores were available for both timepoints for 101 participants with no disabilities, 45 participants with one or more disabilities, and 30 participants with VI (Table 5).

**Table 5:** Within-group comparison of T1 and T2 PSQI global and component scores by subgroup.

		No disability			1+ disabilities			VI		
		*T1*	*T2*	Wilcoxon-signed rank test	*T1*	*T2*	Wilcoxon-signed rank test	*T1*	*T2*	Wilcoxon-signed rank test
Global sleep quality	*n*	101			45			30		
	*M*	6.12	7.02	*T* = **2,494**, *p* < **0.01**	8.69	9.62	*T* = 565, *p* = 0.079	7.80	8.50	*T* = 232.5, *p* = 0.293
	*SD*	3.62	3.84		4.93	5.13		4.66	4.75	
	*Mdn*	6.00	7.00		8.00	8.00		6.50	7.50	
	*IQR*	6	6		7	10		7	7	
Sleep duration	*n*	104			48			32		
	*M*	0.31	0.55	*T* = **465**, *p* < **0.01**	0.85	1.02	*T* = 119.5, *p* = 0.106	0.81	0.88	*T* = 27, *p* = 0.564
	*SD*	0.68	0.83		1.05	1.19		1.03	1.10	
	*Mdn*	0.00	0.00		0.00	0.50		0.00	0.00	
	*IQR*	0	1		2	2		2	2	
Sleep efficiency	*n*	104			48			32		
	*M*	1.23	1.51	*T* = **999**, *p* < **0.05**	1.65	1.92	*T* = 137, *p* = 0.225	1.44	1.81	*T* = 64, *p* = 0.187
	*SD*	1.29	1.25		1.38	1.33		1.41	1.42	
	*Mdn*	1.00	1.00		2.00	3.00		1.00	3.00	
	*IQR*	3	3		3	3		3	3	
Sleep latency	*n*	104			47			32		
	*M*	1.19	1.07	*T* = 625.5, *p* = 0.195	1.38	1.45	*T* = 176, *p* = 0.418	1.19	1.19	*T* = 82.5, *p* = 0.761
	*SD*	1.04	1.02		1.13	1.14		1.09	1.06	
	*Mdn*	1.00	1.00		1.00	1.00		1.00	1.00	
	*IQR*	2	2		2	2		2	2	
Sleep disturbance	*n*	103			49			33		
	*M*	1.23	1.32	*T* = 300, *p* = 0.117	1.69	1.76	*T* = 99, *p* = 0.513	1.70	1.64	*T* = 18, *p* = 0.564
	*SD*	0.51	0.51		0.65	0.78		0.64	0.78	
	*Mdn*	1.00	1.00		2.00	2.00		2.00	1.00	
	*IQR*	1	1		1	1		1	1	
Sleep quality	*n*	105			49			33		
	*M*	1.01	1.20	*T* = **589**, *p* < **0.05**	1.37	1.43	*T* = 99, *p* = 0.513	1.21	1.30	*T* = 63, *p* = 0.467
	*SD*	0.66	0.80		0.95	0.79		0.89	0.77	
	*Mdn*	1.00	1.00		1.00	1.00		1.00	1.00	
	*IQR*	0	1		1	1		1	1	
Use of sleep medication	*n*	104			48			32		
	*M*	0.24	0.34	*T* = 116.5, *p* = 0.165	1.00	0.98	*T* = 44, *p* = 0.915	0.97	0.88	*T* = 14, *p* = 0.571
	*SD*	0.70	0.82		1.37	1.34		1.36	1.31	
	*Mdn*	0.00	0.00		0.00	0.00		0.00	0.00	
	*IQR*	0	0		3	3		3	3	
Daytime dysfunction	*n*	104			49			33		
	*M*	0.89	1.01	*T* = **319.5**, *p* < **0.05**	1.24	1.53	*T* = **193**, *p* < **0.05**	1.12	1.42	*T* = **85**, *p* < **0.05**
	*SD*	0.67	0.65		0.80	0.89		0.78	0.87	
	*Mdn*	1.00	1.00		1.00	1.00		1.00	1.00	
	*IQR*	1	0		1	1		1	1	

*n*, number of participants with valid T1 and T2 scores; *M*, mean score; *SD*, standard deviation; *Mdn*, median score; and *IQR*, interquartile range. Statistically significant results are shown in bold.

**Figure 1:**
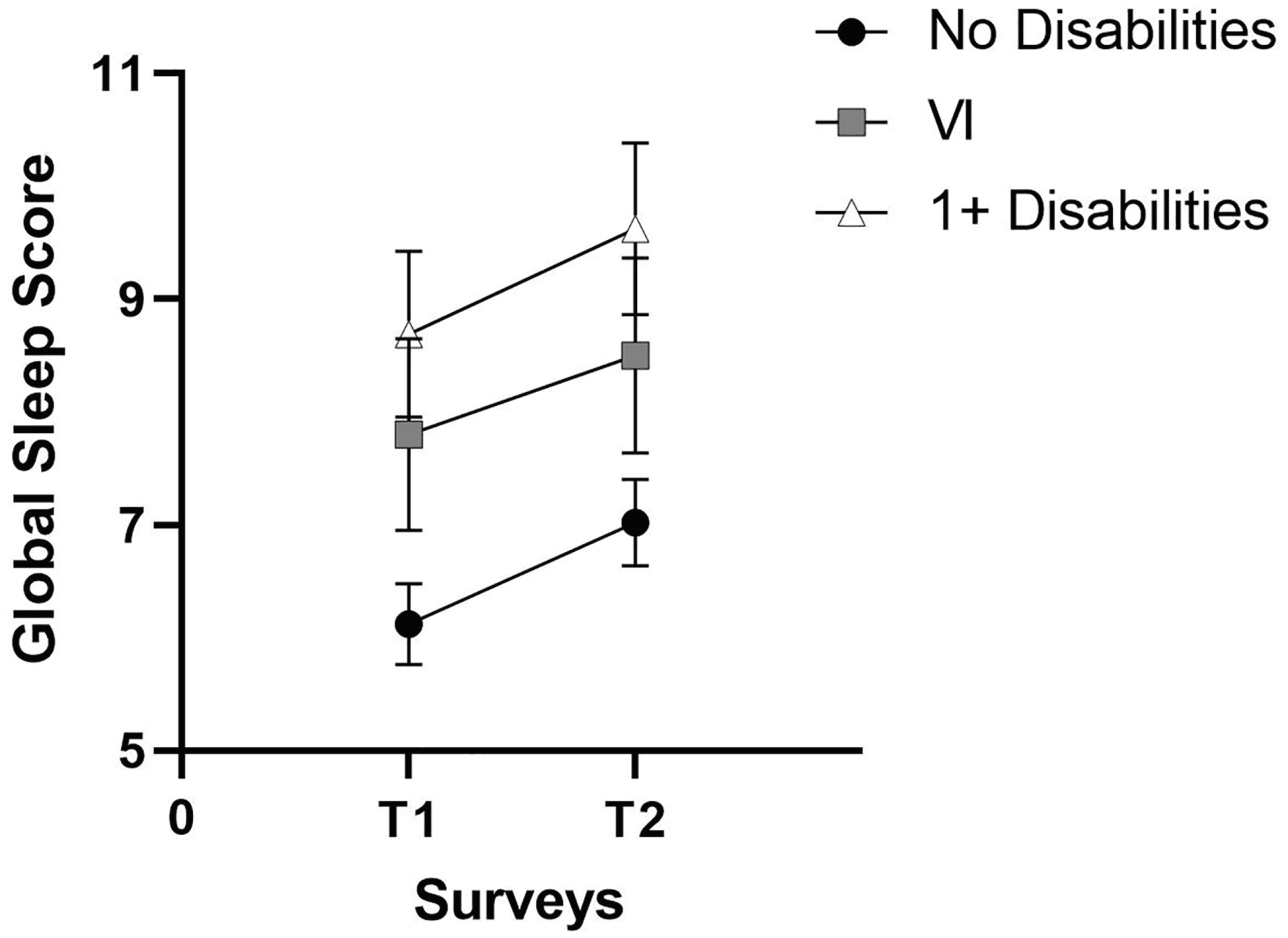
Pittsburgh Sleep Quality Index global sleep mean scores at T1 and T2.

**Figure 2:**
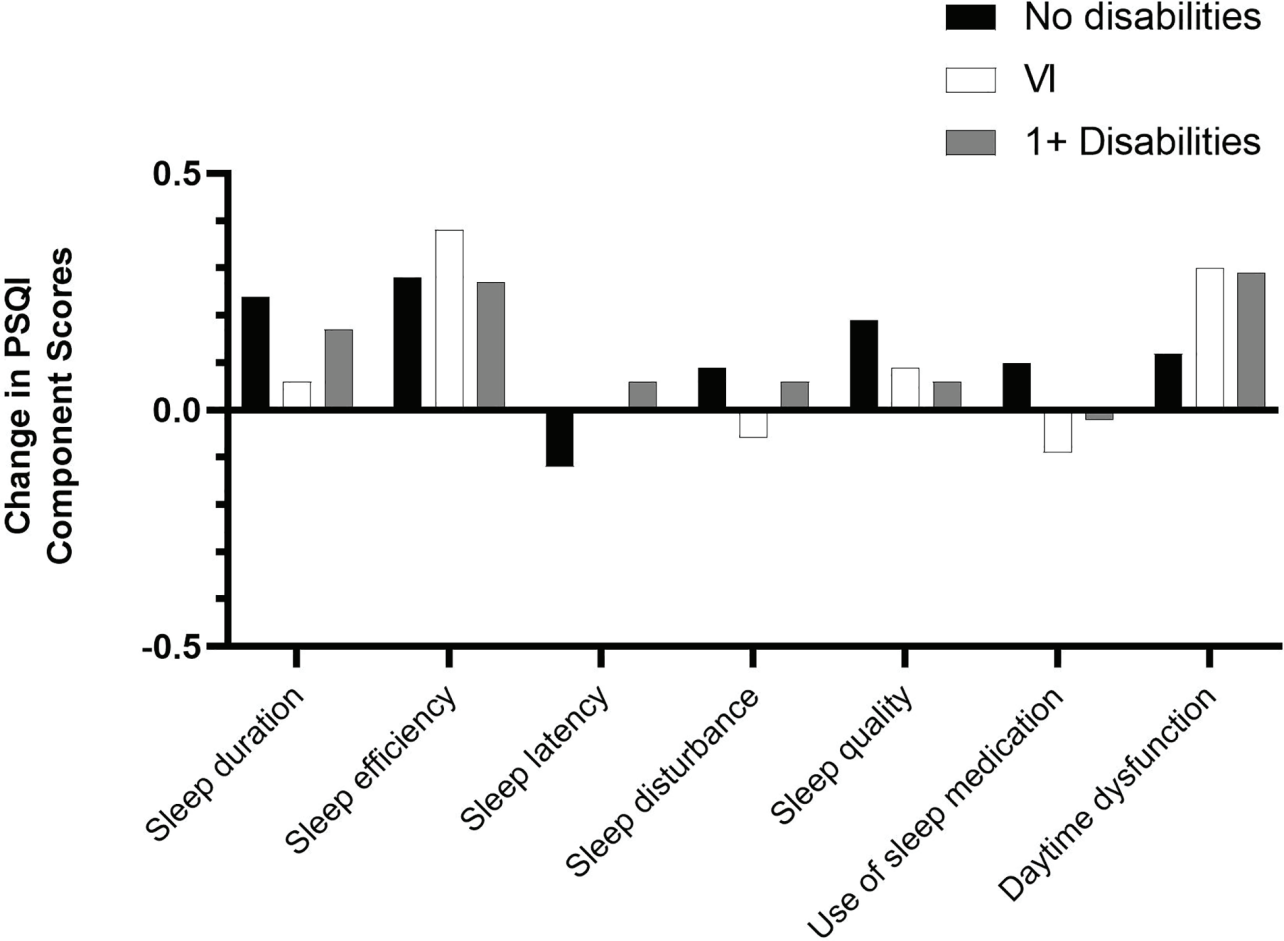
Change in PSQI component mean scores.

There were no statistically significant changes in median global sleep quality and six of the component scores between T1 and T2 within participants with one or more disabilities, except for a statistically significant increase in daytime dysfunction. Aside from a small decrease in the use of sleep medication, the mean scores for global sleep quality (Figure 1) and the remaining six PSQI components all increased (Figure 2). The biggest increases in this group were observed for daytime dysfunction, sleep efficiency, and sleep duration, with the proportion of participants who reported getting <5 h of sleep increasing from 11.8% at T1 to 18.0% at T2.

Similar trends were found when focusing on the VI group consisting of participants with VI only and those with VI and comorbid conditions. There were no statistically significant differences in median global PSQI scores and six of the seven PSQI component scores over time, but, as for the group of participants with one or more disabilities, there was a statistically significant increase in daytime dysfunction. Furthermore, mean scores increased for global PSQI sleep quality (Figure 1) and four of the seven PSQI components except for sleep latency, sleep disturbance, and use of sleep medication (Figure 2). The largest increase in this group was seen in sleep efficiency, while the proportion of participants with VI who rated their sleep quality as “very good” fell from 20.0% at T1 to 11.4% at T2.

In contrast, participants with no disabilities reported significantly poorer global sleep quality, sleep duration, sleep efficiency, self-reported sleep quality, and daytime dysfunction at T2. Mean scores increased across all seven component scores except for sleep latency in this group (Figure 2), with the biggest mean increases observed for sleep efficiency, sleep duration, and self-reported sleep quality. For instance, the proportion of participants without disabilities getting 7 or more hours of actual sleep decreased from 79.0% at T1 to 61.5% at T2, while the proportion of those getting <5 h of sleep increased from 2.9% to 5.8%. Similarly, 6.7% rated their sleep quality as “very bad” in T2 compared to none at T1. In contrast, the proportion rating their sleep as “very good” fell from 21.0% at T1 to 17.1% at T2.

### Predictors of Sleep Quality

A hierarchical linear regression was run to determine whether the addition of disability (having one or more disabilities vs. having no disabilities) predicted sleep quality when controlling for age and gender in the first step, and factors previously associated with sleep quality (see Table 6) in the second step. The full model of gender, age, state anxiety (revised), loneliness, self-isolation, alcohol consumption, and disability (Model 3) was statistically significant, *F*(7, 473) = 49.41, *p* < 0.001; adjusted *R*^2^ = 0.414. The addition of state anxiety (revised), loneliness, self-isolation, and alcohol consumption in Model 2 explained an additional 37.4% of the variance in sleep quality above and beyond age and gender, *F*(6, 474) = 49.32, *p* < 0.001. The addition of disability in Model 3 accounted for an extra 3.8% of the variance in sleep quality. Higher levels of anxiety, loneliness, and having one or more disabilities significantly contributed to explaining sleep quality in the final model.

**Table 6:** Hierarchical multiple regressions for PSQI sleep quality.

	Variable	T1		T2	
		*B*	*β*	*B*	*β*
Model 1	Constant	9.036^***^		11.512^***^	
	Age	−0.033^*^	−0.101	−0.081^**^	−0.237
	Sex	0.031	0.003	1.194	0.131
Model 2	Constant	−1.703		2.488	
	Age	0.011	0.034	−0.038	−0.111
	Sex	−0.328	−0.037	0.086	0.009
	Anxiety (revised)	0.151^***^	0.472	0.138^***^	0.423
	Loneliness	0.059^***^	0.182	0.056	0.174
	Self-isolation	0.246^*^	0.076	0.096	0.073
	Alcohol	−0.300^**^	−0.097	−0.266	−0.076
Model 3	Constant	−2.939^**^		1.436	
	Age	−0.011	−0.033	−0.054^*^	−0.157
	Sex	0.100	0.011	−0.561	−0.062
	Anxiety (revised)	0.146^***^	0.454	0.139^***^	0.427
	Loneliness	0.047^**^	0.145	0.038	0.119
	Self-isolation	0.200	0.061	0.054	0.042
	Alcohol	−0.199	−0.064	−0.193	−0.055
	Disability	2.168^***^	0.224	2.136^**^	0.220
		Model 1	Model 2	Model 3		
T1	*R* ^2^	0.010	0.384	0.422		
	*F*	2.52	49.32^***^	49.41^***^		
	*ΔR* ^2^	0.010	0.374	0.038		
	*ΔF*	2.52	71.98^***^	31.14^***^		
T2	*R* ^2^	0.051	0.381	0.414		
	*F*	3.90^*^	14.58^***^	14.21^***^		
	*ΔR* ^2^	0.051	0.331	0.033		
	*ΔF*	3.90^*^	18.96^***^	7.82^**^		

*N_T1_* = 481; *N_T2_* = 149. *B* = unstandardized regression coefficient; *β* = standardized coefficient; *R*^2^ = coefficient of determination; *ΔR*^2^ = adjusted *R*^2^; and *ΔF* = *F* change.

* *p* < 0.05;

** *p* < 0.01;

*** *p* < 0.001.

The procedure was repeated for T2 to determine whether the factors identified at T1 consistently predicted sleep quality (see Table 6). The full model of gender, age, state anxiety (revised), loneliness, self-isolation, alcohol consumption, and disability (Model 3) was statistically significant, *F*(7, 141) = 14.21, *p* < 0.001; adjusted *R*^2^ = 0.385. The addition of anxiety, loneliness, self-isolation, and alcohol consumption in Model 2 explained an additional 33.1% of the variance in sleep quality above and beyond age and gender, *F*(6, 142) = 14.58, *p* < 0.001; adjusted *R*^2^ = 0.355. The addition of disability in Model 3 accounted for an extra 3.3% of the variance in sleep quality. Being younger predicted sleep quality at T2. As at T1, higher levels of anxiety and having one or more disabilities significantly contributed to explaining sleep quality in the final model but, unlike T1, loneliness did not.

## Discussion

This paper set out to provide a preliminary assessment of sleep quality over time in individuals with disabilities, with a focus on those living with VI, during the COVID-19 pandemic. Overall, sleep quality was found to be consistently poorer in participants with disabilities, including those with VI, than in participants with no disabilities. Although it accounted only for a small amount of variance, disability emerged as a consistent predictor of sleep quality across both timepoints when controlling for age, gender, and other factors previously associated with sleep quality, such as alcohol consumption (Romero-Blanco et al., 2020; Robillard et al., 2021), anxiety (Xiao et al., 2020), and self-isolation (Pérez-Carbonell et al., 2020). Individuals with disabilities scored significantly worse across all seven PSQI components than those with no disability at T1 (April–May 2020), reflecting existing evidence of comparatively poorer sleep in individuals with a disability during the pandemic (Fancourt et al., 2021).

Previous research has found that people with VI often report poor sleep quality and greater sleep-related complaints than those without a VI (Tabandeh et al., 1998; Zizi et al., 2002; Tamura et al., 2016; Peltzer and Phaswana-Mafuya, 2017). In the current study, global sleep quality was consistently poorer in individuals with VI than those with no disability; however, the difference between the two groups was no longer statistically significant at T2. Reflecting existing evidence (Tabandeh et al., 1998; Peltzer and Phaswana-Mafuya, 2017), individuals with VI also reported shorter sleep duration, increased sleep latency, more disturbed sleep, and increased use of sleep medication compared to individuals with no disability during the early stages of the pandemic. By T2 (March 2021), only sleep disturbance and use of sleep medication remained significantly poorer in those with VI. Furthermore, except for daytime dysfunction, there was no significant deterioration in overall sleep quality nor in any of the PSQI components for those with VI and those reporting any type of disability. This contrasts with the significant deterioration in sleep quality identified in participants without disabilities and suggests that the pandemic may have had a greater impact on the sleep of individuals with no disabilities. One possible reason for this may be that self-isolation and experiences of loneliness are not necessarily new for people living with disabilities, which impact mobility and social contact (Brunes et al., 2019). The majority of people with disabilities commonly have comorbid disabilities and health conditions (Havercamp et al., 2004; Barnett et al., 2012; Court et al., 2014), which may have resulted in greater health concerns prior to the pandemic. Thus, the impacts of worries relating to health, self-isolation, and/or limited social contact on sleep may have been greater amongst those for whom these concerns were novel.

Secondly, given evidence of the impact of VI on sleep before the pandemic (Tabandeh et al., 1998; Tamura et al., 2016; Peltzer and Phaswana-Mafuya, 2017), the negative impacts of the pandemic on sleep may not be as apparent among this group compared to those without a disability, whose sleep may have been comparatively better prior to the pandemic. Indeed, around two-thirds of participants with VI in this study were categorized as poor sleepers at both timepoints. This is comparable to the proportion reported elsewhere for visually impaired people with no light perception (65.6%) but higher than that reported for those with light perception (45.8%; Tabandeh et al., 1998). In contrast, around 56% of participants with no disabilities were categorized as poor sleepers in the current study, a figure substantially higher than the 9.1% reported for controls without VI by Tabandeh et al. (1998). Baseline figures for sleep quality, social contact, and experiences of self-isolation prior to the pandemic were not available in the current study, and therefore, the reasons behind the different sleep experiences of individuals with and without disabilities can only be postulated.

Contrary to previous research, the current study did not find an association between self-isolation and sleep (Pérez-Carbonell et al., 2020). It is possible that feelings of loneliness experienced as a result of self-isolation, rather than self-isolation itself, impact sleep, although loneliness predicted sleep quality only at T1. Levels of loneliness were significantly higher in participants with disabilities and VI than those with no disabilities at both timepoints, and although not statistically significant in any of the three groups, bigger increases in loneliness were observed in participants with disabilities and VI (Heinze et al., 2021). Further research is required to confirm the impact of loneliness on sleep. A ceiling effect may be one possible explanation, with the impact of loneliness on sleep reducing as feelings of loneliness become increasingly normalized by the individual. Once again, this may reflect a greater impact of restrictions on social contact in people without disabilities, for whom loneliness may have been a novel experience at T1. In addition, being younger predicted sleep quality at T2 but not at T1. This contradicts previous findings which associated older age with poorer sleep quality (Gadie et al., 2017). In contrast, state anxiety was a significant predictor of sleep quality across both timepoints and accounted for a large proportion of the variance in sleep quality. This supports existing evidence, which points to the negative impact of anxiety on sleep (Altena et al., 2020; Xiao et al., 2020; Evans et al., 2021; Robillard et al., 2021; Villadsen et al., 2021). In this sample, state anxiety was consistently higher in participants with disabilities and VI, although statistically significant differences between those with and without disabilities were found at T2 only (Heinze et al., manuscript submitted for publication). Given associations between disability and anxiety (Sareen et al., 2006; Brenes et al., 2008; Kempen et al., 2012), these findings have important implications for the design of interventions targeted at improving sleep quality for individuals with disabilities beyond the pandemic. State anxiety may be an essential factor to consider in any such intervention.

### Limitations and Future Directions

The current study highlights a number of important findings relating to sleep quality in people living with disabilities such as VI during the COVID-19 pandemic. However, some limitations must be acknowledged. Firstly, due to convenience sampling, and recruitment of participants through professional and personal networks within the sight loss sector, extrapolation of findings to the general population cannot be made. Additionally, the sample size and number of valid scores for T2 were considerably smaller than for T1. Thus, longitudinal comparisons relied on a smaller subsample than was available at both timepoints. Secondly, findings relating to sleep quality in people with VI should be interpreted with caution. Due to small sample sizes in T2, it was not possible to control for comorbid disabilities, which may have impacted on sleep. Future research is needed to assess both sleep quality in people living with VI only, and the relationship between disabilities other than VI and sleep quality. Exploration of the potential differences in sleep between those who report one, and those who report multiple, comorbid disabilities, would also be valuable.

Participants were recruited from the membership of Blind Veterans UK, a charity which provides its members with access to support relating to sleep and health, including targeted sleep hygiene interventions. The survey was also promoted through other sight loss organizations, which may have increased sleep education and sleep quality among respondents. Future research should collect information on the support that participants have accessed relating to sleep and consider how this support may mediate sleep experiences during, and following, the easing of COVID-19 restrictions.

Next, while the majority of participants resided in the United Kingdom at both timepoints, responses were received from as many as 22 different countries at T1. Measures implemented to tackle the pandemic, and public information campaigns, may have differed substantially between these countries. Due to small sample sizes, it was not possible to provide geographical comparisons of sleep experiences, but research in this area may provide useful insights into the impacts of national policy on this aspect of public health and help to inform best practice and future policy.

Finally, the current study reports on findings relating to two surveys undertaken during the COVID-19 pandemic, a period characterized by its impact on everyday life, work, and social experiences. The period following restriction easement may offer a similarly novel range of experiences and challenges, which may impact on aspects of health and well-being, including sleep. Future research is needed to explore individuals’ sleep experiences during this transition period and beyond, to establish the long-term health implications of the pandemic, particularly among individuals living with VI and/or other disabilities.

## Conclusion

The current paper provides a preliminary assessment of sleep quality in people with disability during the COVID-19 pandemic, with a focus on those living with VI. It offers insight into the factors, which may have played a role in sleep quality during the COVID-19 pandemic, including not only disability and VI, but also other health and social factors. While sleep was consistently poorer for individuals with disabilities, including those with VI, the pandemic appeared to have a greater impact on individuals with no disabilities, who experienced a significant deterioration in their sleep over time. State anxiety and, to a lesser degree, disability were consistent predictors of sleep quality at both timepoints, and interventions designed to alleviate sleep difficulties should seek to address the role of state anxiety in sleep quality.

## Data Availability Statement

The datasets presented in this article are not readily available because participants were not asked if they consented to their data being shared outside the research team involved in this study as part of the consent process. Requests to access the datasets should be directed to renata.gomes@bravovictor.org.

## Ethics Statement

Ethical review and approval was not required for the study on human participants in accordance with the local legislation and institutional requirements. The patients/participants provided their written informed consent to participate in this study.

## Author Contributions

NH designed and performed the analysis and wrote the paper. SH wrote and edited the paper. CC designed the survey, wrote the paper, and edited the paper. LG-M consulted on data analysis and edited the paper. TK designed the survey and produced graphics for the paper. SF advised on survey design and data analysis and reviewed the paper. CE advised on survey design and reviewed the paper. RG designed the survey and edited the paper. All authors contributed to the article and approved the submitted version.

## Funding

This study was funded by Blind Veterans UK.

## Conflict of Interest

The authors declare that the research was conducted in the absence of any commercial or financial relationships that could be construed as a potential conflict of interest.

## Publisher’s Note

All claims expressed in this article are solely those of the authors and do not necessarily represent those of their affiliated organizations, or those of the publisher, the editors and the reviewers. Any product that may be evaluated in this article, or claim that may be made by its manufacturer, is not guaranteed or endorsed by the publisher.

## Abbreviations

PSQI: Pittsburgh sleep quality index
STAI-S: State anxiety subscale of the state-trait anxiety index
T1: Timepoint 1 (survey 1)
T2: Timepoint 2 (survey 2)
VI: Visual impairment
